# What, what for and how? Developing measurement instruments in epidemiology

**DOI:** 10.11606/s1518-8787.2021055002813

**Published:** 2021-07-29

**Authors:** Michael Reichenheim, João Luiz Bastos

**Affiliations:** I Universidade do Estado do Rio de Janeiro Instituto de Medicina Social Hésio Cordeiro Departamento de Epidemiologia Rio de JaneiroRJ Brasil Universidade do Estado do Rio de Janeiro. Instituto de Medicina Social Hésio Cordeiro. Departamento de Epidemiologia. Rio de Janeiro, RJ, Brasil; II Universidade Federal de Santa Catarina Departamento de Saúde Pública FlorianópolisSC Brasil Universidade Federal de Santa Catarina. Departamento de Saúde Pública. Florianópolis, SC, Brasil

**Keywords:** Epidemiologic Measurements, Data Accuracy, Cross-Cultural Comparison, Validation Studies as Topic

## Abstract

The development and cross-cultural adaptation of measurement instruments have received less attention in methodological discussions, even though it is essential for epidemiological research. At the same time, the quality of epidemiological measurements is often below ideal standards for the construction of solid knowledge on the health-disease process. The scarcity of systematizations in the field about what, what for, and how to adequately measure intangible constructs contributes to this scenario. In this review, we propose a procedural model divided into phases and stages aimed at measuring constructs at acceptable levels of validity, reliability, and comparability. Underlying our proposal is the idea that not only some but several connected studies should be conducted to obtain appropriate measurement instruments. Implementing the model may contribute to broadening the interest in measurement instruments and, especially, addressing key epidemiological problems.

## INTRODUCTION

Considered one of the pillars of public health, epidemiology is chiefly concerned with the frequency, distribution, and determinants or causes of health events in human populations^1^ . By emphasizing these aspects, the measurement of related events — either dimensions of the health-disease process or factors that are causally related to it — is key in the development of research in the field. Epidemiologists employ considerable efforts to measure specific health-disease conditions, assess characteristics (of person, place, and time) that allow establishing comparisons and assessing variability, as well as address the processes underlying their occurrence in a given population domain^2^ . Although there are exceptions, the epidemiological measurement of these processes and factors is predominantly quantitative, which allows the subsequent statistical analysis of their patterns of association in order to assess the health event and intervene upon it.

The measurement process is not a trivial activity. Rather, it is of considerable complexity and imposes important challenges. This process implies expressive conceptual rigor, in addition to the other issues discussed in greater detail further on in this article^6 , 7^ . It is impossible to measure —within acceptable levels of validity and reliability— a phenomenon of epidemiological interest predicated on an ambiguous definition, be it among researchers or even in the population whose health-disease conditions are the object of study. The use of instruments with good psychometric properties is equally important to measure aspects of interest in a given population^6^ . In their absence, not only validity and reliability of the measurements become questionable, but it is also more challenging to compare data across studies on the same health event^8^ , limiting the proper construction of scientific knowledge on the research object. Knowledge is often established by the systematic accumulation and contrasting of research results that, by assumption, must be amenable to confrontation.

Albeit essential to any epidemiological research, measurement has received less emphasis in the methodological discussions pervading the field. While issues related to study design, potential biases, and statistical techniques often guide epidemiology courses and debates, relatively less space has been allocated to rigors and processes related to measurement. In this scenario, the need for a comprehensive assessment is clear, which includes the stages of theoretical construction and formal psychometric tests employed in the development or adaptation processes of measurement instruments. The authors of this paper were unable to find a discussion about the differences between what, what for, and how a measurement instrument should be developed—including the evaluation of its internal and external structures. The aim of this review is, therefore, to offer a set of guiding principles on possible paths to be followed for the development or cross-cultural adaptation of measurement instruments used in epidemiological studies. By proposing a procedural model comprised of sequential phases and stages, our expectation is that this study will contribute to improve the quality of knowledge production in the health field. We also hope that it will improve academic training in epidemiology. Encouraging the acquisition of information from the specific area of measurement can help students and researchers develop skills and competencies necessary to adhere to the proposal.

Our stance is eminently indicative, though, as the literature on the subject matter is complex and vast. We chose to focus on only a few points with immediate relevance and applicability to epidemiological practice. We used only widely recommended bibliographic references; some specific publications are also cited as suggestions to guide particular processes or decisions. We hope that this introduction will encourage broader readings on the covered topics.

## RESEARCH SCENARIOS AND INSTRUMENT DEVELOPMENT OR ADAPTATION

Epidemiological studies require well-defined and socially relevant research questions, which, in turn, demand reliable and accurate measurements of the phenomena and concepts needed to answer them^8^ . Berry et al.^9^ discuss three perspectives that are particularly relevant for the issues at hand.

From the absolutist perspective, sociocultural nuances are disregarded in the interpretation of health-related events, thus assuming the possibility of unrestricted comparisons between quantitative measurements carried out across populations. In this case, a single measurement instrument could be widely employed in different populations, and results could be directly compared to consolidate scientific knowledge about the object of interest.

The relativist approach lies in a diametrically opposite position. Accordingly, sociocultural specificities are placed at the forefront, so that a different measurement instrument should be used for each population. This approach denies the possibility of quantitatively comparing measurements taken in socioculturally differentiated populations, since instruments would not be equivalent to each other, and the only way to contrast them would be through qualitative analyses.

The universalist perspective assumes an intermediate position, implying both the quantitative measurement of investigated phenomena and the possibility of comparisons between populations. This stance recognizes sociocultural nuances and the need to acknowledge them. If there is similarity in the way events are interpreted among different populations, it would be possible to pursue a so-called “universal” instrument, albeit adapted to each particular situation. According to this view, cross-cultural adaptation would ensure equivalence across different versions of the same instrument^10^ . Its application would allow socio-culturally distinct populations to be quantitatively compared, based on equivalent measures of the same problem of interest.

The universalist approach^3 , 6 , 11 , 12^ implies three possible scenarios, which must be evaluated and identified by the investigator when selecting the research instrument to be used in the study:

- there is an established and adapted instrument for use in different populations, including the population of interest (Scenario 1);
- an instrument is available, but it requires additional refinement, given its limited applicability to the population of interest, either because it requires complementary psychometric assessments, or because it still needs to be cross-culturally adapted (Scenario 2); or
- no instrument is available and it is necessary to develop an entirely new one (Scenario 3).

In Scenario 2, cross-cultural adaptation studies are often needed, in which the concept of equivalence is a guiding principle^10^ . Equivalence is usually unfolded in conceptual, item, semantic, operational, and measurement equivalence: all need to be assessed to consider an instrument as fully adapted. In Scenario 3, the researcher should adjourn the original research initiative and develop completely original instruments^13^ . In this case, undertaking a parallel research program to develop an instrument capable of measuring the parameters of interest is necessary. This is crucial, since conducting the research without good measurement instruments puts the whole project at risk; it decreases the chances of contributing to the advancement of knowledge or of attending to a particular health need, becoming, thus, ethically reprehensible. Most of the time, epidemiological studies are conducted within the limits of Scenarios 2 and 3, the first being the most common in the Brazilian research context.

An important implication of working within these scenarios is the need to know, in detail, the state of the art of the available instruments. Such knowledge is essential for adapting, refining, or developing measurement tools.

## PHASES OF DEVELOPMENT OR ADAPTATION OF INSTRUMENTS

Different procedural stages need to be followed whether the objective is to develop a new instrument or cross-culturally adapt an existing one. The Figure shows one proposed procedural model. The first phase elaborates and details the construct to be measured, which involves several steps: specifying, preparing, and refining the items regarding their empirical and semantic contents; detailing operational aspects, inter alia, the scenarios under which the instrument will be administered; and implementing several pre-tests to refine some aspects, such as item wording and their understanding by the target population. Provisionally called “prototypical” because it involves assembling one or more sketches of the instrument (i.e., prototypes or preliminary versions) to be subsequently tested, this first phase of the process is essential for achieving good results. This step is as essential in the development of a new instrument as in cross-cultural adaptations, in which the notion of equivalence (referred to in the previous section) requires thorough examination. This must be emphasized, since the efforts dedicated to this stage are often scarce in adaptation processes—if not completely ignored.

**Figure:**
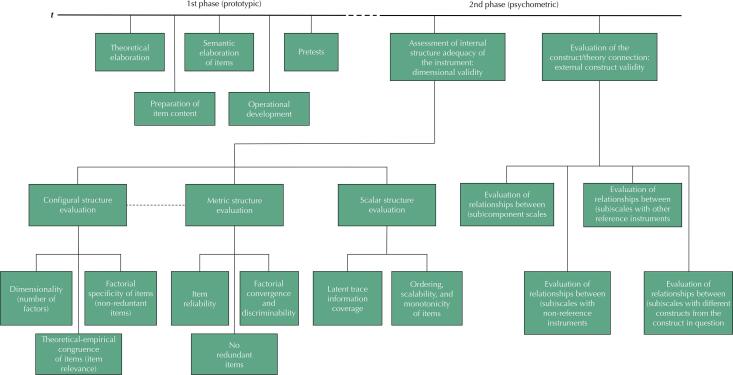
Proposal of a procedural model for the development or cross-cultural adaptation of an instrument.

Besides its importance in achieving a functional instrument, this first phase is not only essential from a substantive point of view—in the search for correspondence between the construct to be measured and the tool for doing so—but it also makes the next phase, testing of prototypes, more efficient. Enough time allocated for this stage and procedural rigor decrease the possibility of finding problems in subsequent validation studies, which are generally large and, therefore, more expensive. The worstcase scenario is to find pronounced deficiencies at the end of a long and intricate process, involving multiple interconnected studies, and having to go back to the field to test an almost new prototype.

The prototype specified in the previous phase is then examined in an extensive second phase, which we would call “psychometric.” Unlike the first one, in which qualitative approaches are more prominent, this second phase, as already suggested, comprises a sequence of larger quantitative studies. Expanding the upper right part of the Figure , the lower portion shows the various psychometric aspects of this phase. Two distinct segments should be noted: one concerns the internal structure of the instrument, covering its configural, metric, and scalar structures; and the other addresses its external validity, assessing whether patterns of association between estimates derived from the instrument and measures of other constructs agree with what is theoretically expected, for example.

Before detailing the proposed procedural model, it is worth mentioning the types of instruments to which the Figure refers. As should be clear throughout the text, the model we propose involves constructs (dimensions) in which the object of study intensifies or recedes according to a particular gradient. Although these types of constructs are common—e.g., diseases and injuries, depression, psychosocial events (violence), perceptions of health or quality of life—in some cases this increasing or decreasing severity or intensity is not applicable or does not matter much. A good example is what we might call “inventories”: a questionnaire to investigate whether an individual was ever exposed to a given chemical agent. Here, the instrument should contain a wide range of questions about potential contact situations over a period, with just one endorsement (hit) required to confirm the respondent’s exposure. Although one can think of a second instrument to capture the degree of exposure to this chemical agent—measuring the increasing intensity of this exposure—such an inventory would not focus on an underlying gradient. Another situation in which the model in the Figure would not be applicable refers to pragmatic instruments based on a set of risk predictors that are not theoretically linked to an underlying construct. An example would be a tool to predict the risk of dying from Covid-19 at the first contact of a patient with the health service, composed of variables covering several aspects, such as sociodemographic characteristics, health-related behaviors, pre-existing conditions, recent contacts with Covid-19 cases, or even admission exams. Though extremely important, this set of items would still not constitute a construct to be mapped.

In many other situations, the items of an instrument do not connect with a construct and/or form an explicit gradient of intensity. It is, therefore, up to the researcher to assess them and to evaluate whether a procedural model—such as the one proposed here—applies to the problem at hand. The following three sections provide some details about the two phases. It is worth pointing out that there are numerous paths to be followed and that our choice is only one of many. To the interested reader, we suggest checking the related bibliography; in the following section, we discuss some of it.

## PROPOSING AN INSTRUMENT

The details of the first phase of the procedural model illustrated in the Figure can be found in Box 1 . Adapted from Wilson’s^13^ proposal, the process contains five distinct stages. In the first, the theory supporting the construct is evaluated as to the extent to which it represents what one wants to measure. This representation is technically called the “construct map,” which outlines the ideas the developers (or adapters) of the instrument have about what is to be captured, including its gradient of intensity^13^ . The construct map guides the process of developing items that will reflect the construct in question. The aim is to arrive at an efficient and effective set of items with good measurement properties. The goal is, at the end of the process, to identify those items that—in the most discriminating and orderly way possible—can map the metric space of the construct. Consisting of items positioned in the expected increasing gradient of intensity^13^ . The empirical expression of the construct map is sometimes called the Wright map, which consists of the selected items positioned in the expected increasing gradient of intensity.

**Box 1:** Prototypical phase: aspects to evaluate in the proposition of an instrument.

Evaluation stage	Description and purpose	Questions to be answered	Technique/method/model	Empirical expression
Evaluation of the theory upon which the construct is based	Theoretical appreciation of the construct that one desires to assess, in relation to both a potential multidimensionality and a gradient of intensity in each dimension. This stage develops the construct map (dimensional)^13^.	What is the definition of the construct of interest? Are there postulated subdimensions for the construct? Which are they? What would be the theoretical elements of this dimension(s) and how would they be organized in a gradient of intensity?	Literature review. Consultation with experts.	There is no empirical expression of this aspect, since the definition of the construct, its gradient of intensity, and its possible subdimensions are fundamentally theoretical questions.
Item content evaluation	Identification of the empirical manifestations of the dimension(s) and how they cover parts of the construct map. In this stage, a preliminary content validity (a.k.a. face validity) is proposed, connecting the empirical expression of the item content to the underlying theoretical elements.	Do items have contents tied to the underlying dimension? Are the items distinct from each other in terms of content? Is each part of the construct map represented by a specific item? Do the items cover the construct map sufficiently and adequately (i.e., without gaps and/or occupying a similar position to other items)?	Literature review. Consultation with experts. Qualitative approaches with members of the target population (in-depth interviews, focus groups, etc.)^36,37^.	Individually, each item reflects a specific part of the construct map. Together, the items should sufficiently and adequately cover the contents of the underlying construct (or, in case it is multidimensional, each constituent dimension).
Item semantics specification	Writing items to better convey their content to the respondent.	Do the terms used in writing up the items allow item allow its direct and unambiguous connection to specific parts of the construct map?	Consultation with linguistics experts and experts on the subject matter, as well as translators (in the case of adaptations)^6,11^.	Items of the instrument and their specific writing.
Evaluation of operational aspects	Assess and decide on how the instrument is to be administered—face-to-face interviews, self-completed forms, computer-assisted questionnaires etc.—which includes assessing the adequacy of the operational scenario. In this stage of the process, an evaluation of the contribution of each item to the construct map begins, including consideration of levels/categories of the outcome.	What is the most appropriate mode of administration, considering the target population? In what operational scenario should the instrument be administered?	Consultation with experts and members of the target population via qualitative studies^36,37^.	Mode(s) of application of the instrument in the desirable operational scenario. Any instrument should be evaluated in light of a preestablished operational scenario, preferably early on in its development process (or adaptation process).
Pre-tests (including preliminary reliability tests)	Medium-sized studies (e.g., n = 100–150) aimed at evaluating: Acceptance, understanding, and emotional impact of the items. Formal aspects related to the sequence of items or rules for skipping them. Instrument response options, *vis-à-vis* the operational scenario (operational aspects). This stage can also be used for preliminary reliability analyses, focusing on internal consistency, inter- and intra-observer agreement/test-retest etc.	Does the instrument have an acceptable degree of understanding? Are the reactions aroused by the items in the respondents within what was expected? Does the sequence of items contribute to an easy administration for interviewers and/or respondents? Are the response options in line with respondents’ ability to discern them? Does the operational scenario favor the interaction between instrument and respondent, or interviewer and respondent? Are there indications of good reliability in preliminary studies (pre-tests)?	Administration of the instrument in the target population, possibly including alternative formulations of the items. A sequence of studies should be carried out until one or more prototypes are obtained for the second phase of instrument development (or adaptation)^3,6^.	Records of the administration of the instrument in the target population. Reliability indicators (acceptability differs by type). See Reichenheim et al.^6^ for more details. See also Streiner et al.^7^, Nunnally and Bernstein^38^, Raykov and Marcoulides^39^, Price^40^ and Shavelson and Webb^41^.

Note: References: Streiner et al.^7^, Beatty et al.^42^, Moser and Kalton^43^, Bastos et al.^3^, Reichenheim and Moraes^6^, Johnson and Morgan^44^, DeVellis^45^; Gorenstein et al.^46^ Some of these references are occasionally marked, when necessary, along with other specific ones.

Moving from the theoretical-conceptual dimension of the construct map to the empirical dimension of the items requires contextualization and, thus, a good grasp of the population to which the instrument will be administered. On the one hand, the construct (and what it represents within the underlying theory) should be pertinent to the population in question. On the other hand, eligible items must be potentially endorsed in the desired context. It is always necessary to ask whether an item has some potential to be endorsed or whether negative answers to it do not stem from an intrinsic impossibility. As an example, we can mention an item on explicit discrimination experienced in the workplace asked to schoolchildren who still have not reached working age. Although somewhat obvious when pointed out, this is a common problem that requires careful consideration.

Once the construct map is specified, it is used to identify and develop the items that will be part of the instrument. At this stage, researchers should identify the various ways in which the construct manifests, including its different levels of intensity^13^ . Box 1 distinguishes the process of identifying items from how these will be conveyed to respondents. These are, indeed, different tasks. The process of identifying potential items derives directly from the construct map, having to do with recognizing the empirical manifestations representing the outlined gradient of intensity. It concerns the content (meaning) of an item and not its form (wording). Syntactic and semantic questions come in later (third step), when the number of candidate items have been further restricted through sequential qualitative studies^3 , 6^ .

The fourth stage of the first phase concerns operational issues, starting with the specification of the outcome space of each item. Identifying the type and number of response categories that items should contain is an important task. Like other eminently operative issues—instrument format, administration scenario etc.—debating and specifying the types of answers should be done early on, as soon as the target population of the instrument is identified. The third stage is then resumed with this focus: writing the qualifications of the response categories that were previously outlined/defined.

At this point, it is worth emphasizing that the validity of an instrument—its adequacy and performance—is dependent upon a close connection with the background content, attention to respondents’ cognitive and emotional capacity, and a productive environment in which answers can be provided with ethics, spontaneity, and safety. One should keep in mind that even a validated instrument can still underperform if administered to a population for which it was not originally developed or in an adverse operative context.

Item design and outcome specification require a first visit to the target population so that the first batches of prototypes (i.e., alternative and preliminary versions of the instrument) are assessed regarding acceptability, understanding, and emotional impact. A good strategy is to pre-test the instrument (fifth stage). Based on evidence from the pre-test, the most promising prototypes are then put to test in the next phase. Box 1 provides additional information and suggests several references for consultation.

## ASSESSING INTERNAL STRUCTURE ADEQUACY

As already shown in the Figure , Box 2 expands the second phase of the development or adaptation of instruments: the structures to assess (configural, metric, and scalar); the properties under evaluation and the main questions to be answered; the models and analytical techniques used; as well as comments on what is expected of each property and how to evaluate it, including the demarcations guiding decisions.

**Box 2:** Psychometric phase 1: assessing the internal structure adequacy.

Structure to evaluate	Property under evaluation	Questions to be answered	Model(s)^a,b^/parameter(s)	Comments
Configural	(Assumed) Dimensionality	Does the configural structure assumed in the first phase (“prototypic”) arise? Can it be supported?	PCA, EFA/ESEM, CFA. Preliminary eigenvalues, followed by the number of emerging factors in factor analyses.	One expects that the proposed dimensionality in the previous phases will be corroborated; otherwise, it is worth exploring alternative dimensional structures. From a preliminary PCA perspective, this can be observed through the number of emerging > 1.0 eigenvalues. When the ratio between the first and second eigenvalue is greater than four, some authors suggest the possibility of unidimensionality.^47^ Going further with CFA, the amount of dimensions is evaluated through internal diagnosis suggesting poor configural specification (e.g., using Modification Indexes and Expected Parameter Changes via Lagrange Multiplier tests^17,19^). In the case of an analysis with ESEM^48^, it is possible to observe directly alternative structures beyond those theoretically assumed.
	Theoretical relevance of items (theoretical-empirical congruence)	Do the items really belong in their respective dimensions, based on the results of the analysis?	EFA/ESEM and/or CFA. Positioning or location of items in factors.	The items should express their respective factors, distinct from each other, as planned in the instrument development or adaptation process. If any item manifests dimensions other than those theoretically predicted, it must be revised.
	Factor specificity	Is each item linked to only one dimension? Is there ambiguity?	EFA/ESEM and/or CFA. Cross-loading items.	If an item contains factorial specificity, the factor loading should not present ambiguity. The item is expected to be a unique expression of the factor it supposedly represents. Items violating this property should be identified and, depending on the situation, modified, or even replaced.
Metric	Reliability/discrimination of items	What is the magnitude of the relationship between the items and the factors that underlie them?	EFA/ESEM and/or CFA/IRT. Item loadings and residuals.	For the item to be considered reliable, its factorial loading should be above a pre-specified demarcation. The literature does not stipulate a particular value. Conventionally, 0.30^17,49^, 0.35^50^, or 0.40^51^ are considered acceptable cut-off points to admit an item as reliable. Reliability is also tied to the notion of discriminability, since factor loadings are related to IRT parameters, which express the discrimination of an item. By plotting curves from different a_i_ (corresponding to λ_i_), it is possible to visualize them in the Item Characteristic Curve and then make a decision.
	Absence of redundancy of item content	Do items overlap in such a way that they do not map the construct independently?	ESEM, CFA/IRT. Residual correlation (implying violation of conditional/local independence).	In principle, it is expected that items of a given factor show no residual correlations. They are expected to be independent, once conditioned to the factor they supposedly reflect. Violation of independence implies that the variability of the items has another common source, in addition to the factor they represent. The magnitude of a residual correlation—from which a conditional independence violation can be inferred—is somewhat arbitrary. One possibility is to choose a theoretically sustainable value or level (for example, 0.20 or 0.25) and statistically compare models with or without the estimated residual correlation. Another possibility is to follow recommendations from authors to guide the decision-making process. Reeve et al.^53^ suggest the simple demarcation of ≥ 0.3 to admit the existence of residual correlation. Some demarcations are based on formal statistics. One is the Chi-square-based local dependence (LD χ^2^),proposed by Chen e Thissen^54,55^, which uses the ≥ 10 cut-off point to indicate dependence. Another is the Q3 statistic (and variants), as suggested by Yen^56–58^. Several situations lead to correlation between item residuals (errors)^59^, but a common process in instrument development (or adaptation) refers to the presence of content (partial) redundancy between items (in general, pairs). Theoretical evaluation—observing semantics, and denotative and connotative meanings of the respective contents—should be sought when a statistical violation is observed.
	Convergent factorial validity (CFV).	Do the items convergently reflect the corresponding factor?	CFA. Average Variance Extracted (AVE)	CFV refers to each factor, as its name implies. It is understood that CFV occurs if the relationship between the AVE of the items—i.e., the variance that the items have in common—is at least greater than the joint variance of the respective errors, which express item variability due to other factors. Thus, quantitatively, the CFV is endorsed if the AVE ≥ 0,5^17,60^. From an interpretative perspective, endorsing CFV means accepting that the dimension (factor) in question is “well attended” by the respective set of items, since they contain more factor information than error (from sampling and/or measurement/process and/or inherent to the components^61^). A related indicator— √ *AVE* —summarizes the construct reliability (dimension). Thus, values ≥ 0.7 also indicate convergence and, strictly, that it is internally consistent (i.e., consistency of/between items, internal to the factor to which they belong)^60^.
	Discriminating factorial validity (DFV)	Is the amount of information captured by the set of items in their respective factors greater than that shared among the component factors (discriminant)?	CFA. Contrast of the average variance extracted (by the items) of a given factor with the square of the correlations of this factor with the others of the system.	This property only applies to multidimensional constructs. If there is DFV, a larger information “flow” is expected from the factors to the items than between the factors themselves. Demarcation of DFV violation may follow some generic rule of thumb or a more formal evaluation. Some authors suggest factorial correlations of 0.80 to < 0.85 as indicative of violation and ≥ 0.85 as violation per se^17^. A more rigorous strategy is to formally test the statistical significance of the difference between the AVE of the factor and the square of its correlations with others^60^. A positive and statistically significant sign of this difference would endorse DFV, while a statistically significant negative sign would favor its rejection, indicating violation. A nonsignificant positive or negative difference may be an indication for or against a violation. On a more conservative stance, a violation could be based only on a statistically significant difference.
Scalar	Coverage of latent trait information (by each item and the set of items).	Does the item set cover most of the latent trait or are there “unmapped” regions? In the latent trait regions effectively mapped, are the items evenly distributed or are there clusters indicating redundancy?	Parametric IRT. Eyeballing, using the Wright Map, which consists of combining the construct map with estimates of the item placement obtained in the IRT and chart observation analyses.	It is expected that items will be able to properly position individuals (or any other unit of analysis) along the construct map. The spectrum of variation predicted by the construct map should also be covered appropriately. One way to evaluate these two aspects is to critically assess the position of the items according to the proposed Wright Map^13,27^. In this sense, the correspondence of item positioning is considered along the latent spectrum—for example, via b_i_ parameters obtained in IRT analyses—and the increasing intensity presented in the construct map^13^. This eyeballing procedure should be followed by an analysis of the information coverage^21,62^. Specific charts allow you to indicate whether the set of items covers most of the latent trait or if there are regions with gaps (without items). These graphs also help detect whether all latent trait regions are effectively covered, whether items are distributed evenly, or if there are clusters, indicating overlap and positioning/mapping redundancy. Additional graphic evaluations allow, in a complementary way, to assess the behavior of the items, especially regarding the latent trait coverage. Obtained by parametric IRT, these graphs include the Item Information Functions and Item Characteristic Curves. When items are polytomous, the Category Characteristic Curves are obtained. They also serve to evaluate the items “internally,” observing the coverage areas of each level and whether they are ordered according to the theoretical assumption of the construct map. Examples of these graphs can be found in the references cited at the end of this Table or in Internet searches (https://www.stata.com/manuals/irt.pdf).
	Ordering according to item stability or monotonicity.	Do items mapping regions of the construct map do so in the theoretically expected order of intensity or are there regions of the construct wherein less severe (lighter/milder) items supplant other items that, in principle, should be capturing more intense areas of the latent trait?	Nonparametric and parametric IRT. Loevinger’s H, Mokken criterion and graphic assessments.	The items should separate well the regions of the latent trait (content)—area that they supposedly cover—avoiding overlapping as much as possible. Two strategies allow checking this property: ordering according to scalability and monotonicity. Ordering items according to scalability refers to the coherence between the frequencies with which the items are endorsed and the part of the construct map that they should cover. In an ideal scenario, it is expected that a respondent with low intensity of a given latent trait of the construct (dimension) effectively endorses a representative item (mapper) of this region of “lower” intensity, while not endorsing another item that reflects a more intense degree of the construct. This aspect can be analyzed by item and by the whole set of the instrument. Loevinger’s H coefficient reflects this^63–65^. With the value 1.0 as the upper limit of adequacy, an estimate of at least 0.3 is recommended for the set of items^64,66^. An H below this value indicates an instrument with poor scalability. According to Mokken^66^, values of 0.3 to < 0.4 indicate weak scalability; 0.4 to < 0.5, average; and ≥ 0.5, strong scalability. In an acceptable instrument, most of the H estimates of each item should also follow these references. The assumption of monotonicity is another related property to be appreciated during the evaluation of scalar behavior of each item and, by extension, of the set formed by them^64,65^. Monotonicity can be supported when the probability of confirmation positive of an item increases according to the increase in intensity of the latent trait. Visually, there is a violation of simple monotonicity when the probability of endorsement declines as the total (latent) score grows. Additionally, a violation of double monotonicity occurs if there is any crossing along the curves of the items obtained in a IRT analysis. Whether single or double, monotonicity is present when the criterion suggested by Mokken is < 40^66^, understanding that some item crossings can be attributed to the sample variability. Values between 40 and 80 serve as a warning, demanding a more detailed evaluation by the researchers; a criterion higher than 80 raises doubts about the monotonicity hypothesis of an item, as well as the scale as a whole^63,64^.

^a^ Legend: ACP - principal component analysis; CFA - confirmatory factor analysis; AFE - exploratory factor analysis; ESEM - exploratory structural equation modeling; IRT - item response theory; CFV - convergent factorial validity; DFV - discriminating factorial validity; AVE - average variance extracted.

^b^ References: Gorsuch^67^, Rummel^68^, Brown^17^, Kline^19^, Marsh et al.^48^, Embretson and Reise^62^, Bond and Fox^27^, De Boeck and Wilson^69^, Van der Linden^21^, Davidov et al.^30^ Some of these references are occasionally marked, when necessary, along with other specific ones.

Box 2 highlights how many properties need to be scrutinized before judging the internal structure as adequate, thus endorsing this validity component of the instrument^15 , 16^ . This is at odds with the general literature on the topic, in which the validity of an instrument tends to be accepted by somewhat sparse and weak evidence. Quite often, decisions on the acceptability of the instrument rely on a few factor analyses, using only model fit indices, demarcated by generic cut-off points (e.g., Root Mean Square Error of Approximation/RMSEA, Comparative Fit Index/CFI, Tucker-Lewis Index/TLI^17^ ). These analyses usually fall short in further examining items and the scale(s) as a whole. Strictly speaking, the range of properties listed in Box 2 does not fit in single products (e.g., scientific articles), and serial studies are often necessary to visit one or more properties at a time. The methodological intricacies relating to each property certainly require detailing and greater editorial space.

A previously addressed point illustrates this fundamental rigor: the need for explicit demarcations to decide whether an item or scale meets the property under scrutiny. All estimators used in the evaluations require specific cut-off points, so that choices can be replicated or, when appropriate, criticized, rejected, or modified during the development or adaptation of an instrument. Box 2 offers some landmarks indicated in the literature. Beyond prescriptive benchmarks, these should serve as a stimulus to the empirical examination of an instrument. The main point is that the many decisions related to the psychometric suitability of an instrument need clear anchors, previously agreed upon by peers of the scientific community. The literature would certainly be enriched if these details extended to scientific articles.

One point to make regarding the procedural context in question is that multivariate analyses are used as diagnostic devices. As process tools, they must answer the central questions posited *a priori* . In this sense, it is necessary to distinguish eminently qualitative from quantitative issues related to a technical and methodological sphere. The third configural property presented in Box 2 serves as an example. Rather than simply verifying whether an exploratory factor analysis identifies cross-loadings, it is important to answer if a violation of factorial specificity effectively occurs, which would be antithetical to what was projected in the first phase of instrument development. A cross-loading suggests ambiguity in the item, and therefore that its clear-cut function as an “empirical representative” of the dimensional construct map was not fulfilled. Here, quantitative evidence meets qualitative findings, signaling a problem and the need for action, either by modifying the item semantics, or by replacing it with an item with better properties. The other properties demand the same approach.

In addition to the internal properties of items and scales summarized in Box 2 , two other related questions deserve mentioning. The first concerns the presumption of measurement invariance (configural, metric, and scalar) ^17^ . The assumption that the instrument performs similarly in different population subgroups is almost a rule. Often, it is tacitly assumed that the instrument functions equally well across groups (e.g., genders, age groups, strata with different levels of education or residing in different parts of the country), so that any differences observed between them are considered factual and not due to measurement problems. However, without further evidence, this is a difficult argument to sustain since inconsistent functioning of an instrument among subgroups of the population can lead to incorrect inferences and inefficient or even harmful health decisions and actions^20^ . This demands stepping up research programs on measurement instruments. Beyond scrutinizing their properties, evaluating them in various population segments is also needed. To ensure invariance of the instrument in different population subgroups is to allow reliable comparisons.

Along with invariance is the issue of equalization and linking of instruments^22^ . These concern the search for common metrics across instruments that supposedly capture the same construct, but hold different items and/or varied response options^25 , 26^ . In both cases, one must be careful when summarizing and comparing studies. Study results may not be comparable—even if focused on the same construct—when they are conducted in different populations and with different instruments. Without equalization, measurement instruments may lack metric and scalar tuning.

An issue related to the scalar properties of an instrument concerns the appropriateness of grouping individuals when applying cut-off points to scores (whether crude scores, formed by the sum of item scores, or model-based scores, such as factor-based or Rasch scores^27 , 28^ ). This point deserves attention, especially regarding the approaches frequently used in epidemiology. It is common to categorize a score into a few groups, by taking the mean, median, or some other “statistically interesting” parameter as a cut-off point. This procedure has downsides, however, since the study population is not necessarily partitioned into internally homogeneous and externally heterogeneous groups. Substantive knowledge on the subject matter is undoubtedly crucial in the process of grouping respondents appropriately, but the search for internally similar yet comparatively distinct groups may gain from using model-based approaches, such as latent class analyses or finite mixture models^29^ .

## ASSESSING CONNECTIONS BETWEEN CONSTRUCTS AND THEORIES

Box 3 proposes a typology that is in line with validity based on hypothesis testing presented in the early 2010s by the COSMIN initiative (COnsensus-based Standards for the selection of health Measurement INstruments)^15 , 16 , 33^ . Contrary to the apparent conciseness of the suggested typology, this stage of the second phase of instrument evaluation implies a long process—perhaps as long as the study of the construct itself in all its relationships of causes and effects. Evoking other texts^7 , 11^ , it is important to point out that the validity of an instrument ultimately corresponds to establishing validity of the theoretical construct that the instrument aims to measure. Somewhat circular and dismaying due to the long road it projects, this reasoning alerts us to how risky and reckless it is to support an instrument that has been assessed by only a few studies. Consolidating and eventually endorsing the suitability of an instrument requires many tests, both regarding its internal structure and its external connections.

**Box 3:** Psychometric phase 2: Assessing connections between constructs and theories

Evaluation stage	Questions to be answered	Technique/method/model^a^	Comments^a^
Evaluation of relationships between the (sub)scales of the instrument.	Are the (sub)scales of the instrument associated in the expected direction and magnitude?	Parametric or nonparametric association tests between the (sub)scales of the instrument.	This aspect could have already been contemplated in the assessment of discriminant validity involving factorial correlation, in the stage of evaluation of the internal structure. At this moment of analysis, however, the tests are already based on the scale scores themselves (whether crude or model-based), refined in previous stages, mainly regarding scalar structure.
Evaluation of relationships between (sub)scales with other instruments of the same construct that are not considered reference	Does the instrument associate with another one that measures the same construct in a similar (convergent) way? At what magnitude?	Comparison of extreme groups and parametric or nonparametric association tests.	This stage concerns construct validity. Together, construct, content, and criterion validity are known as the three Cs described in many textbooks on classical measurement theory.
Evaluation of relationships between (sub)scales with another instrument (or procedure) considered reference for the construct itself.	Is the instrument capable of measuring what is proposed when there is another one regarded as reference?	Estimation of sensitivity, specificity, and area under the ROC (Receiver Operating Characteristic) curve of the instrument, based on a concurrent criterion (reference instrument) and/or a predicted (future) outcome.	The literature traditionally calls this stage as criterion validity (one of the three Cs), subdivided into concurrent and predictive validity.
Evaluation of relationships between the (sub)scale with others outside of the construct in question.	Does the instrument confirm the general predictions and hypotheses of the theory that involves it, i.e., its nomological network? Is the instrument unrelated to other constructs that are not part of the general theory that encompasses the phenomenon of interest?	Multivariate data analysis, complex causal models, and other statistical techniques that allow analysis of relationships of interest with greater rigor and accuracy.	Evaluation of relationships between the (sub)scale with others outside of the construct in question.

^a^ References: Streiner et al.^7^, Bastos et al.^3^, Reichenheim and Moraes^6^, Lissitz^70^, Armitage et al.^71^, Corder and Foreman^72^, Kline^19^, Little^61^, Hernán and Robins^5^, VanderWeele^35^.

As suggested in Box 3 , external validation of an instrument ranges from simple tests of association between component subscales to intricate hypotheses tests about the construct—what scholars often take as the nomological network of interconnected concepts of a theory^5 , 7 , 34 , 35^ . Whatever the level of complexity of the study, a question that arises—often in the context of scientific publications—is *when* an external validity study should be performed, given all the necessary prior steps to better know the intricacies of the instrument. Would it be worth conducting studies along the lines that Box 3 indicates, without first having some evidence about the sustainability of the instrument’s configural, metric and scalar structures? One should recognize that correlations between scales (e.g., the instrument in question and others that cover the same construct) may well occur even in the face of multiple psychometric insufficiencies at the internal level. What would these correlations mean, knowing, for example, that the set of items does not satisfactorily meet the requirements of factorial specificity, convergent factorial validity, and scalability? The answer based on the mere correlation would indicate external validity, but one could ask “of what?” if the ability to represent the underlying construct is flawed and uninformative. These questions cannot be answered clearly, but it is necessary to pose them before “blindly” carrying out external validity studies. The timing of these stages is a decision to be taken within each research program, but the saying “haste makes waste” serves as a reminder: little time and effort (and resources!) invested in one step can be double time and effort (and resources!) needed in a following step.

## CONCLUDING REMARKS

This article clarifies that the development of a measurement instrument involves an extensive process, comprising multiple connected studies. This trajectory can be even longer and tortuous considering the need for replication studies or when certain psychometric studies raise fundamental questions that only returning to the prototypic phase of development may provide answers. This panorama contrasts sharply with the way epidemiologists often approach measurement instruments. Contrary to common practice, evidence on the adequacy of a measurement tool demands more than one or two studies on its dimensional structure, or the magnitude of factor loadings. This warning also extends to critical analyses of external validity that, as mentioned in the former section, require attention to the inner workings of the instrument.

The development and refining of different versions of the instrument are also vital, so that research carried out in distinct populations retains comparability and can be compared with each other. The cross-cultural adaptation process is as intricate as the development of a new instrument. All phases and stages apply equally to adaptation processes. In fact, a researcher performing a cross-cultural adaptation often finds a variety of gaps in the original research program giving rise to the instrument. Sometimes, they are problems related to the execution of previous studies; other (many) times, several properties have not even been assessed. In this case, the focus shifts from equivalence (see section on Research Scenarios) to the core of the structure of the instrument. This is not trivial, since the origin of the problem is always dubious: an intrinsic problem of the instrument or a problem in the process of cross-cultural adaptation. Be that as it may, examining an instrument in another sociocultural context requires even more time and effort. That is why many consider cross-adaptation as an additional construct validation step^33^ .

A recurring question is whether all phases and stages need to be completed to deem an instrument suitable for research or for use within health services. This question is difficult to answer, but some milestones may guide us. One suggestion has already been offered in the section on the process stages: a well-planned and developed prototypic phase helps greatly to obtain favorable results in the second major phase of the process. Rigor in the first phase contributes to better psychometric properties; it also adds efficiency, as several problems tend to be solved or even avoided early on. Epidemiological studies in the psychometric phase are usually large and, therefore, rarely susceptible to replications to solve emerging issues.

Another guide is resorting to the fundamentals: always remembering the essence of each property and what its violation means. For example, would we firmly declare an instrument as valid and ready for use in light of a few exploratory factor analyses—preliminary stating a configural structure—and/or some studies correlating the score(s) of the (sub)scale(s) with certain sociodemographic variables as evidence on theoretical pertinence? Given the range of the substantive and procedural possibilities, would this be sufficient, or should we postpone the use of the instrument and obtain additional evidence to support its validity? We reiterate that a quick and prompt response does not exist, but that, perhaps, a rule can be useful for decision-making: even if we are not prepared to let the great mess the good—or even let the good get in the way of the reasonable—it may be worth letting the reasonable get in the way of the bad. Although this is a subjective perspective, always negotiable among peers, if put into practice it will possibly lead us to better instruments and, as we have already pointed out, to better results and comparisons between studies or health interventions.

The continuous development, refinement and adaptation of measurement instruments should be an integral part of epidemiologic research. Knowledge construction requires instruments with acceptable levels of validity and reliability, up to par with the level of rigor commonly required in the elaboration of study designs and their complex analyses. Meticulousness and rigor in these spheres are pointless if the dialogue between publications and appreciation of consistent scientific evidence fail due to precariousness of measurement instruments. As products focused on collective use, measurement instruments require development processes that resemble those found for medicines or other health technologies. And as such, they deserve care and dedication.
