# Supplementary material for: What, what for and how? Developing measurement instruments in epidemiology
Source: Rev Saude Publica. 2021 Jul 29;55:40. doi: 10.11606/s1518-8787.2021055002813 (PMC8323826; doi:10.11606/s1518-8787.2021055002813)
Supplement: Translation

# Translation: O quê, para quê e como? Desenvolvendo instrumentos de aferição em epidemiologia

**Authors:** Michael Reichenheim, João Luiz Bastos

**Affiliations:** I Brasil Universidade do Estado do Rio de Janeiro. Instituto de Medicina Social Hésio Cordeiro. Departamento de Epidemiologia. Rio de Janeiro, RJ, Brasil; II Brasil Universidade Federal de Santa Catarina. Departamento de Saúde Pública. Florianópolis, SC, Brasil

**Keywords:** Medidas em Epidemiologia, Confiabilidade dos Dados, Comparação Transcultural, Estudos de Validação como Assunto

## Abstract

Embora fundamental para a pesquisa epidemiológica, o desenvolvimento e a adaptação transcultural de instrumentos de aferição têm recebido menos destaque nas discussões metodológicas que permeiam o campo. Em paralelo, a qualidade das mensurações realizadas em muitos estudos epidemiológicos está frequentemente aquém do desejado para a construção de conhecimento sólido sobre o processo saúde-doença. A escassez de sistematizações sobre o que, para que e como aferir na área provavelmente contribui para esse cenário. Nesta revisão, propomos um modelo processual composto por uma sequência de etapas voltadas à mensuração de construtos em níveis aceitáveis de validade, confiabilidade e, por extensão, comparabilidade. Subjaz à proposta a ideia de que não apenas alguns, mas diversos estudos concatenados entre si e sucessivamente mais aprofundados devem ser conduzidos para obter aferições adequadas. A implementação do modelo poderá contribuir para alargar o interesse sobre instrumentos de aferição e, especialmente, para enfrentar os problemas investigados em epidemiologia.

## INTRODUÇÃO

Considerada um dos pilares da saúde pública, a epidemiologia se preocupa fundamentalmente com a frequência, a distribuição e os determinantes ou as causas de eventos de saúde em populações humanas^1^ . Ao enfatizar esses aspectos, a atividade relacionada à mensuração dos fenômenos de interesse – sejam eles dimensões do processo saúde-doença ou questões que o condicionam – assume centralidade nas pesquisas desenvolvidas na área. O epidemiologista emprega parte considerável de seus esforços na mensuração de condições específicas de saúde-doença, de características (de pessoa, lugar e tempo) que permitam observar sua variabilidade e de processos subjacentes à sua ocorrência em um determinado domínio populacional^2^ . Embora haja exceções, a mensuração epidemiológica dos aspectos mencionados é predominantemente quantitativa, o que permite a subsequente análise estatística de seus padrões de associação com vistas a apreciar o evento de saúde e intervir sobre ele^3^ .

Ainda que central à epidemiologia, o processo de mensuração não consiste em uma atividade trivial. Pelo contrário, é de considerável complexidade, impondo importantes desafios a serem enfrentados. Tal processo implica expressivo rigor conceitual, além das outras questões discutidas em maior detalhe neste artigo^6 , 7^ . Não é possível mensurar com níveis aceitáveis de validade e confiabilidade um fenômeno cuja definição é ambígua entre os pesquisadores ou a própria população cujas condições de saúde-doença são objeto de apreciação. Igualmente importante é o uso de instrumentos com boas propriedades psicométricas para mensurar os aspectos de interesse na população^6^ . Na ausência destes, não somente a validade e a confiabilidade das mensurações são potencialmente postas em xeque, como também fica mais difícil comparar os dados com os de outras pesquisas acerca do mesmo evento de saúde^8^ , limitando, por sua vez, a construção própria do conhecimento científico delimitado no objeto de investigação. Tal conhecimento frequentemente se estabelece pelo acúmulo e contraste sistemático de resultados de pesquisas que, por pressuposto, requerem ser passíveis de confrontação.

Embora fundamental para a pesquisa epidemiológica, há que se reconhecer que a atividade de mensuração tem recebido menos destaque nas discussões metodológicas que permeiam o campo. Enquanto questões ligadas aos desenhos de estudo, aos possíveis vieses e às técnicas estatísticas frequentemente pautam os cursos de epidemiologia e os debates travados entre pesquisadores da área, relativamente menos espaço tem sido destinado aos rigores e aos processos atinentes à mensuração. Neste cenário, observa-se a necessidade de uma apreciação abrangente, que inclua desde as etapas de construção teórica até testes psicométricos formais, empregados no processo de desenvolvimento ou de adaptação dos instrumentos de aferição. Os autores deste estudo não localizaram na literatura uma discussão sobre as diferenças entre o que, para que e como no âmbito do desenvolvimento de um instrumento de aferição, incluindo a avaliação de suas estruturas interna e externa. O objetivo desta revisão é, portanto, oferecer um conjunto de sugestões sobre possíveis percursos a serem seguidos no desenvolvimento ou na adaptação transcultural (ATC) de instrumentos de aferição utilizados em estudos epidemiológicos. Ao propormos um modelo processual composto por uma sequência de etapas, nossa expectativa é que o texto contribua para melhorar a qualidade da produção do conhecimento sobre saúde. Esperamos, também, que o artigo sirva para aprimorar a formação acadêmica em epidemiologia. O incentivo à aquisição de informações na área específica da medição pode estimular estudantes e vindouros pesquisadores a dotar-se das habilidades e competências necessárias para aderir à proposta.

Nossa postura é eminentemente indicativa, uma vez que a literatura sobre o tema é complexa e vasta. Optamos por aprofundar apenas alguns pontos que entendemos de recorte aplicado e de relevância imediata à prática epidemiológica. Nesse movimento, procuramos nos ater a referências bibliográficas amplamente recomendadas, adicionadas de algumas publicações pontuais, como sugestões para demarcar alguma conduta ou decisão específica. Ainda assim, esperamos que esta introdução incentive leituras mais amplas em sequência.

## CENÁRIOS DE PESQUISA E DESENVOLVIMENTO OU ADAPTAÇÃO DE INSTRUMENTOS

Estudos epidemiológicos exigem perguntas suficientemente delimitadas e socialmente relevantes, as quais requerem mensurações confiáveis e acuradas dos fenômenos e conceitos necessários para respondê-las^8^ . Berry et al.^9^ discutem três perspectivas a serem adotadas nesta direção.

Na perspectiva absolutista, desconsideram-se nuances socioculturais na interpretação dos eventos de interesse e assume-se, portanto, que há possibilidade de comparação irrestrita de aferições quantitativas levadas a cabo em diferentes populações. Neste caso, salvo a necessidade de tradução literal para os idiomas pertinentes, um único instrumento de aferição poderia ser amplamente utilizado nas mais variadas populações, podendo-se comparar diretamente os seus resultados com vistas à consolidação do conhecimento científico sobre o objeto de interesse.

Em situação diametralmente oposta encontra-se a abordagem relativista, a qual eleva as especificidades socioculturais à sua máxima importância, pressupondo que um instrumento de aferição diferente seja utilizado para cada nova população investigada. Essa abordagem nega a possibilidade de comparação quantitativa de medidas realizadas em populações socioculturalmente diferenciadas, visto que os instrumentos não seriam equivalentes entre si, e a única forma de contrastá-las seria por meio de análises qualitativas.

Assumindo uma posição intermediária está a perspectiva universalista, que implica tanto a aferição quantitativa de fenômenos em investigação quanto a possibilidade (mas, não garantia) de comparação entre populações distintas por meio dessa medida. Essa posição reconhece as nuances socioculturais e apregoa que devem ser levadas em consideração. Havendo semelhança na forma como os eventos são interpretados em diferentes populações, seria possível prosseguir com a utilização de um instrumento dito “universal”, mas adaptado para cada situação particular. Nessa visão, a ATC garantiria a equivalência entre as suas diversas versões^10^ . Sua aplicação permitiria que populações socioculturalmente distintas fossem comparadas quantitativamente a partir de medidas equivalentes do mesmo problema de interesse.

A abordagem universalista^3 , 6 , 11 , 12^ implica três possíveis cenários, que devem ser avaliados e identificados pelo pesquisador para escolher o instrumental de pesquisa, respondendo se:

- existe um instrumento consagrado e adaptado para uso em distintas populações, incluindo a de interesse (Cenário 1);
- há instrumento disponível, mas seu uso requer cautela ou refinamento adicional, dada sua ainda limitada aplicabilidade à população em tela, seja por necessitar de avaliações psicométricas complementares ou porque ainda precisa ser submetido a um processo de ATC (Cenário 2); ou
- inexiste instrumento, sendo necessário propor o desenvolvimento de um inteiramente novo (Cenário 3).

No Cenário 2, frequentemente há necessidade de desenvolver estudos de ATC, nos quais o conceito de equivalência deve ser tomado como norte^10^ . A equivalência é usualmente desdobrada em conceitual, de itens, semântica, operacional e de mensuração, as quais requerem uma avaliação meticulosa para que se considere um instrumento plenamente adaptado. No Cenário 3, por sua vez, o pesquisador deve suspender a iniciativa original de pesquisa e propor o desenvolvimento de instrumental completamente original^13^ . Aqui, é preciso empreender um programa de investigação paralelo que vise gerar um instrumento capaz de produzir as medidas de interesse. Isso é crucial, uma vez que seguir com a pesquisa sem bons instrumentos de aferição põe todo o projeto a perder, diminuindo suas chances de contribuir com o avanço do conhecimento ou de atender a uma necessidade de saúde, tornando-se, assim, eticamente condenável. Na maioria das vezes, os estudos epidemiológicos são conduzidos nos limites dos Cenários 2 e 3, o primeiro sendo o mais comum e afeito ao contexto brasileiro de pesquisa.

Uma implicação importante de trabalhar em meio a esses cenários é a necessidade de conhecer detalhadamente o estado da arte do desenvolvimento dos instrumentos disponíveis. Tal conhecimento é imprescindível para proceder tanto a uma ATC e/ou ao refinamento de instrumentos de aferição pré-existentes quanto para o desenvolvimento de novo instrumental e a subsequente condução da pesquisa epidemiológica de fundo.

## FASES A PERCORRER NO PROCESSO DE DESENVOLVIMENTO OU ADAPTAÇÃO DE INSTRUMENTOS DE AFERIÇÃO

Seja no caso da proposição de um novo instrumento ou de uma ATC, vislumbram-se etapas processuais distintas, ainda que complementares e interativas. A Figura esquematiza um modelo processual a ser adotado.

**Figura:** Proposta de um modelo processual para o desenvolvimento de um instrumento novo ou de adaptação. transcultural.

A primeira fase visa a elaboração e o detalhamento conceitual do construto a ser medido; a especificação, a confecção e o refinamento dos itens quanto aos seus conteúdos empíricos e semânticos; a pormenorização dos aspectos operacionais, incluindo os cenários de aplicação admissíveis para o instrumento; e várias jornadas de pré-testes para alcançar sintonia fina, como melhorias na redação e na compreensão da população-alvo quanto aos itens. Provisoriamente denominada “prototípica” por encerrar as etapas de construção de um ou vários esboços do instrumento (i.e., protótipos ou versões preliminares) a serem subsequentemente testados, esta primeira fase do processo é essencial para os bons frutos finais. Se, na perspectiva do desenvolvimento de um novo instrumento, esse passo é claramente imperativo, ele não é menos importante em ATC, em que a noção de equivalência (referida na seção anterior) exige um exame minucioso. Este ponto requer ênfase, uma vez que os esforços dedicados às suas fases constituintes são frequentemente parcos nos processos de ATC, quando não totalmente ignorados.

Além de sua central relevância para o alcance de um instrumento funcional, apuro nesta primeira fase não é somente importante do ponto de vista substantivo – na busca de correspondência entre o construto a ser aferido e a ferramenta para sua mensuração –, mas também torna mais eficiente a fase seguinte, de teste dos protótipos. Tempo empenhado e rigor procedimental nesta fase diminuem a possibilidade de se encontrar impropriedades nos estudos de validação subsequentes, que são geralmente de grande porte e, portanto, bastante dispendiosos. O pior cenário é encontrar deficiências marcantes no final de um longo e intrincado processo envolvendo múltiplos estudos encadeados ( *cf* . próxima seção), ter de voltar a fases anteriores de desenvolvimento e retornar ao campo para testar um protótipo praticamente novo.

O protótipo especificado na fase anterior é, então, examinado em uma segunda grande fase, que, também provisoriamente, poderia ser cunhada de “psicométrica”. Diferentemente da primeira, em que preponderam abordagens qualitativas, esta segunda fase, como já aventado, encerra uma sequência de estudos quantitativos de maior porte. Expandindo a parte superior direita da Figura , a porção inferior mostra os diversos aspectos psicométricos que compõem essa fase. Há dois segmentos distintos: um concerne à validade da estrutura interna do instrumento, cobrindo o exame de suas estruturas configural, métricas e escalares; outro aborda sua validade externa, permitindo verificar se o seu comportamento – relativo a medidas de outros construtos, por exemplo, – está de acordo com o que teoricamente se espera.

Antes de passar para o detalhamento do modelo processual proposto, vale uma ressalva sobre os tipos de instrumentos aos quais a Figura se refere. Como deverá ficar claro ao longo do texto, o modelo que propomos envolve, principalmente, construtos (dimensões) em que o objeto em tela, por pressuposto, se intensifica ou remite em gradiente. Ainda que estes tipos de construtos sejam muito frequentes – e.g., doenças e agravos (e.g., depressão), eventos psicossociais (e.g., violências), percepções de saúde ou de qualidade de vida –, há situações em que este (de)crescente de gravidade ou intensidade não se aplica ou não importa tanto.

Um bom exemplo está no que poderíamos chamar de “inventários”, como seria um questionário para investigar se um indivíduo já se expôs a algum agente químico. Aqui, o instrumento deveria conter uma gama de perguntas sobre situações de contato potencial ao longo de um determinado período, sendo o endosso a ao menos uma destas situações a própria positivação do respondente. Ainda que se possa pensar em um segundo instrumento para captar o grau de exposição a este agente químico – e que, portanto, estaria aferindo a intensidade crescente dessa exposição –, para os fins propostos o questionário em tela prescindiria dessa qualidade. Outra situação à qual o modelo na Figura não se aplicaria se refere a instrumentos pragmáticos, baseados em um conjunto de variáveis preditoras de risco que, no entanto, não estariam vinculadas teoricamente a um construto. Um exemplo seria uma ferramenta de predição de risco de letalidade da covid-19 a ser usada ao primeiro contato de um paciente com o serviço de saúde, composta por variáveis que cobrissem diferentes ângulos, como o sociodemográfico, hábitos de vida, condição patológica pregressa, prática preventiva, trajetória e contatos recentes ou, ainda, exames de entrada. Mesmo que claramente de extrema valia, não haveria um construto definido a ser mapeado por esse conjunto.

Há, por certo, muitas outras situações em que itens componentes de um instrumento não se conectam teoricamente e/ou formam um explícito gradiente de intensidade. Compete, pois, ao pesquisador discerni-las e avaliar se um modelo processual como o proposto aqui é pertinente. Alguns pormenores sobre suas duas fases são oferecidos nas três seções seguintes. Vale apontar, no entanto, que as possibilidades são amplas e que, assim sendo, nossa escolha é forçosamente uma de muitas. Ao leitor interessado sugerimos recorrer à bibliografia conexa, da qual oferecemos alguns artigos e livros de interesse no texto que segue.

## ASPECTOS A AVALIAR NA PROPOSIÇÃO DE UM INSTRUMENTO

Os detalhes da primeira fase do modelo processual ilustrada na Figura estão no Quadro 1 . Adaptado da proposta de Wilson^13^ , o processo encerra cinco etapas distintas. Na primeira, avalia-se a teoria que embasa o construto com vistas à representação do que se quer medir. Essa representação é denominada tecnicamente de “mapa do construto”, a qual delineia as ideias que os desenvolvedores (ou, dependendo, os adaptadores) do instrumento têm sobre o que está por ser captado, incluindo seu gradiente de intensidade^13^ . É a partir do mapa do construto que se alicerça a busca dos itens para representá-lo. De muitos possíveis, a proposta é se chegar a um conjunto eficiente e efetivo de itens que contenham boas propriedades de mensuração. A meta é, ao fim do processo, identificar aqueles que, de forma mais discriminante e escalonada possível, consigam mapear o espaço métrico do construto. Constituído de itens posicionados no esperado gradiente crescente de intensidade, o mapa de Wright^13^ é a expressão empírica do mapa do construto.

**Quadro 1:** Fase prototípica: aspectos a avaliar na proposição de um instrumento.

Etapa de avaliação	Descrição e propósito	Questões a serem respondidas	Técnica/método/modelo	Expressão empírica
Avaliação da teoria que embasa o construto	Apreciação teórica do construto que se deseja aferir, tanto em relação a uma potencial multidimensionalidade quanto ao gradiente de intensidade nestas dimensões. Esta é a etapa de desenvolvimento do mapa do construto (dimensional)^13^.	Qual é a definição do construto de interesse? Há subdimensões postuladas para o construto? Quais são elas? Quais seriam os elementos teóricos dessa(s) dimensão(ões) e como se organizariam em um crescente (gradiente) de intensidade?	Revisão de literatura. Consulta a especialistas.	Não há expressão empírica deste aspecto, visto que a definição do construto, seu gradiente de intensidade e suas possíveis subdimensões são questões fundamentalmente teóricas.
Avaliação de conteúdo de itens	Identificação das manifestações empíricas dos componentes da(s) dimensão(ões) e de como estes manifestos cobrem porções do mapa do construto. Nesta etapa se propõe uma validade de conteúdo *a priori* (também conhecido como validade de face), conectando-se a expressão empírica aos elementos teóricos subjacentes.	Os itens do instrumento têm conteúdos próprios e vinculados à dimensão subjacente? Os itens são distintos entre si em termos de conteúdo? Cada porção do mapa do construto está representada por itens específicos? Em seu conjunto, os itens cobrem suficiente e adequadamente o mapa do construto (i.e., sem deixar lacunas e/ou ocupar posição semelhante à de outros itens)?	Revisão de literatura. Consulta a especialistas. Abordagens qualitativas com membros da população-alvo (entrevistas em profundidade, grupos focais etc.)^36,37^.	Individualmente, cada item reflete uma porção específica do mapa do construto. Em conjunto, os itens devem cobrir suficiente e adequadamente o conteúdo do construto subjacente (ou, no caso de este ser multidimensional, cada dimensão constituinte).
Especificação da semântica de itens	Redação dos itens com vistas a maximizar a transmissão de seus conteúdos ao respondente.	Os termos empregados na redação de cada item permitem sua vinculação direta e inequívoca a porções específicas do mapa do construto?	Consulta a especialistas em linguística e no objeto de pesquisa em questão, bem como a tradutores (no caso de ATC)^6,11^.	Itens do instrumento e sua redação específica.
Avaliação de aspectos operacionais	Apreciar e decidir sobre o modo de aplicação do instrumento – por exemplo, face a face, autopreenchimento, eletrônica etc. – na população-alvo, o que inclui avaliar a adequação do cenário de administração. Nesta fase do processo se dá início também a uma avaliação sobre a contribuição de cada item ao mapa do construto, discutindo-se níveis/categorias do desfecho.	Qual é o modo de aplicação mais adequado, considerando a população-alvo de interesse? Em que cenário operacional o instrumento deve ser administrado?	Consulta a especialistas e a membros da população-alvo via estudos qualitativos^36,37^.	Modo(s) de aplicação do instrumento no cenário operacional desejável para sua utilização. Qualquer instrumento deve ser avaliado à luz de um cenário operacional pré-estabelecido, de preferência já no seu processo de desenvolvimento (ou ATC).
Pré-testes (incluindo testes preliminares de confiabilidade)	Estudos de médio porte (e.g., n = 100–150) visando avaliar: Aceitação, compreensão e impacto emocional dos itens. Aspectos formais relativos à sequência dos itens ou regras de pulos. Opções de resposta do instrumento, *vis-à-vis* o contexto de aplicação (aspectos operacionais). Esta etapa também pode ser utilizada para análises preliminares de confiabilidade, focalizando consistência interna, concordância inter e intraobservador/teste-reteste etc.	O instrumento apresenta grau aceitável de compreensão? As reações que os itens provocam nos respondentes estão dentro do esperado? A sequência e o encadeamento dos itens contribuem para uma fácil administração para os aplicadores e/ou respondentes? As opções de resposta estão sintonizadas com a capacidade de discernimento dos respondentes? O contexto de aplicação favorece a interação entre instrumento e respondente ou entrevistador e respondente? Há indícios de boa confiabilidade nos estudos preliminares ( *per* pré-teste)?	Aplicação do instrumento na população-alvo, incluindo, possivelmente, formulações alternativas dos itens. Deve-se executar uma sequência de estudos (jornadas) até se obter um ou mais protótipos a serem utilizados na segunda fase do processo de desenvolvimento (ou ATC) do instrumento^3,6^.	Registros da experiência de aplicação do instrumento a membros da população-alvo. Indicadores de confiabilidade (as demarcações de aceitabilidade diferem segundo o tipo). Consulte Reichenheim et al.^6^para maiores detalhes. Consultar também Streiner et al.^7^, Nunnally e Bernstein^38^, Raykov e Marcoulides^39^, Price^40^e Shavelson e Webb^41^.

* Referências de fundamentação: Streiner et al.^7^, Beatty et al.^42^, Moser e Kalton^43^, Bastos et al.^3^, Reichenheim e Moraes^6^, Johnson e Morgan^44^, DeVellis^45^; Gorenstein et al.^46^Algumas destas referências são também assinaladas ocasionalmente, quando necessário, juntamente com outras específicas.

É preciso entender que o processo de translado do plano teórico-conceitual ao empírico requer contextualização e, assim, uma boa compreensão sobre a população-alvo na qual se intenciona empregar o instrumento em desenvolvimento ou ATC. Por um lado, o construto (e o que este representa no âmbito da teoria subjacente) pressupõe pertinência ao domínio populacional em tela. Por outro, é necessário que os itens elegíveis tenham potencialidade de endosso no contexto previsto. Cabe sempre perguntar se a resposta a um item tem como se realizar e se uma potencial negativação não advém de uma impossibilidade intrínseca. Como exemplo podemos citar um item sobre discriminação explícita vivenciada no domínio laboral perguntado a escolares que ainda não alcançaram o mercado de trabalho. Ainda que um tanto óbvio quando destacado, trata-se de um problema bastante comum e que requer atenção constante.

Uma vez especificado o mapa do construto, passa-se para a identificação e confecção dos itens que comporão o instrumento. É nessa etapa que os pesquisadores deverão identificar as variadas formas pelas quais o construto se manifesta, incluindo suas diferentes intensidades^13^ . De fato, o Quadro 1 distingue o processo de identificação de itens da elaboração de como estes serão transmitidos aos respondentes. São, efetivamente, afazeres distintos. O processo de identificação de potenciais itens deriva diretamente do mapa do construto, tendo a ver com o reconhecimento das manifestações empíricas que representam o gradiente de intensidade esboçado. Diz respeito ao conteúdo (significado) de cada item e não à sua forma (redação). Questões sintáticas e semânticas vêm depois (terceira etapa), quando já se tem um número mais restrito de itens candidatos, selecionados por meio de estudos qualitativos sequenciais^3 , 6^ .

A quarta etapa da primeira fase concerne às questões operacionais, a começar pela especificação do espaço de desfecho de cada item. Identificar o tipo e número de categorias de resposta que cada item deve conter não é algo secundário. Como outras questões eminentemente operativas – formato do instrumento, mídia de veiculação, cenário de aplicação etc. –, debater e especificar o espaço de desfecho dos itens é algo a ser visto precocemente, tão logo seja identificado o público-alvo para o qual o instrumento será direcionado. É com esse foco que, subsequentemente, é preciso retomar a terceira etapa, redigindo-se as qualificações das categorias de resposta antes acordadas no âmbito do conteúdo.

Nesse ponto, vale a pena sublinhar que a validade de um instrumento – sua adequação e seu desempenho – não ocorre em um vazio, mas depende de estreita sintonia com o conteúdo de fundo, da atenção à capacidade cognitiva e emocional dos respondentes e de um ambiente profícuo no qual respostas podem ser oferecidas com ética, espontaneidade e segurança. É preciso lembrar que um instrumento já muitas vezes validado pode ter um desempenho aquém do esperado se for aplicado a uma população para o qual não foi originalmente confeccionado ou em um contexto operativo adverso.

As etapas de desenho de itens e de especificação do espaço do desfecho contemplam uma primeira visita à população-alvo para que os primeiros lotes de protótipos (i.e., versões alternativas e preliminares do instrumento) sejam submetidos a uma avaliação de aceitabilidade, compreensão e impacto emocional. Uma estratégia atraente é pré-testar o instrumento (quinta etapa). A partir das evidências encontradas no pré-teste são escolhidos os protótipos mais promissores, que deverão ser testados formalmente na fase seguinte. O Quadro 1 oferece algumas informações adicionais, bem como sugere diversas referências para consulta.

## AVALIAÇÃO DE ADEQUAÇÃO DE ESTRUTURA INTERNA

Já anunciada na Figura , esta etapa da segunda fase de desenvolvimento ou ATC de instrumentos é aprofundada no Quadro 2 , no qual são apresentados: as estruturas a serem avaliadas (configural, métrica e escalar); as respectivas propriedades em avaliação e as principais questões que demandam resposta; os modelos e as técnicas de análise envolvidos; além de comentários sobre o que se espera de cada propriedade visitada e como avaliá-la, inclusive quanto às demarcações que norteiam decisões.

**Quadro 2:** Fase psicométrica 1: avaliação de adequação de estrutura interna.

Estrutura a avaliar	Propriedade em avaliação	Questões a serem respondidas	Modelo(s)^a,b^/parâmetro(s)	Comentários
Configural	Dimensionalidade (conjeturada)	A estrutura configural proposta na primeira fase (“prototípica”) emerge? Pode ser corroborada?	ACP, AFE/MEEE, AFC. Autovalores, de forma preliminar, seguidos de número de fatores emergentes nas análises fatoriais.	Espera-se que a dimensionalidade proposta nas fases anteriores seja corroborada; caso contrário, cabe explorar estruturas dimensionais alternativas. Na perspectiva de uma análise preliminar, com ACP, isso pode ser observado mediante o número de autovalores > 1,0 emergentes. Quando a razão entre o primeiro e o segundo autovalor é maior do que quatro, alguns autores sugerem a possibilidade de unidimensionalidade^47^. Aprofundando com AFC, o número de dimensões é avaliada por meio de diagnóstico interino sugerindo má especificação configural (e.g., usando Índices de Modificação e Mudanças Esperadas de Parâmetros via Multiplicador de Lagrange^17,19^). No caso de análises por meio de MEEE^48^, é possível observar diretamente estruturas alternativas para além da conjeturada teoricamente.
	Pertinência teórica de itens (congruência teórico-empírica)	A previsão de pertencimento dos itens em suas respectivas dimensões pode ser amparada nos resultados da análise?	AFE/MEEE e/ou AFC. Posicionamento ou localização de itens em fatores.	Os itens devem expressar seus respectivos fatores, distintos entre si, conforme pensado para o instrumento na sua concepção ou em um processo de ATC. Se algum item manifesta dimensões que não aquela prevista teoricamente, há necessidade de revisão.
	Especificidade fatorial	Cada item se vincula a apenas uma dimensão ou não? Há ambiguidade de pertencimento?	AFE/MEEE e/ou AFC. Carga cruzada de itens.	Se um item encerra especificidade fatorial, espera-se que não haja ambiguidade quanto ao carregamento fatorial. Espera-se que o item seja uma expressão exclusiva do fator que supostamente representa. Itens que violam esta propriedade devem ser identificados e, dependendo da situação, modificados ou mesmo substituídos.
Métrica	Confiabilidade/discriminância de itens	Qual é a magnitude da relação entre os itens e os fatores que pressupostamente os manifestam?	AFE/MEEE e/ou AFC/TRI. Carga e resíduo dos itens.	Para que o item seja considerado confiável, espera-se que sua carga fatorial esteja acima de uma demarcação pré-especificada. A literatura não estipula um valor único. Convencionalmente, 0,30^17,49^, 0,35^50^ou 0,40^51^são pontos de corte considerados aceitáveis para admitir um item como confiável. A confiabilidade também se atrela à noção de discriminabilidade, uma vez que cargas fatoriais têm relação direta com os parâmetros *a* obtidos nos modelos TRI, expressando o quão discriminante um item é^52^. Plotando-se curvas de diferentes *a*_i_(correspondentes aos λ_i_), é possível visualizar a discriminância via Curva Característica do Item e decidir.
	Ausência de redundância (particularidade) de conteúdo de itens.	Há itens cujo conteúdo se sobrepõe de tal sorte que não mapeiam o construto independentemente?	MEEE, AFC/TRI. Correlação residual (implicando violação de independência condicional/local).	Em princípio, espera-se que os itens de um determinado fator não mostrem correlações residuais. Espera-se que sejam independentes, uma vez condicionados ao fator que pressupostamente manifestam. Violação de independência implica assumir que as variabilidades dos itens envolvidos se devam a outro motivo comum, para além do tal fator que representam. A magnitude de uma correlação residual a partir da qual se pode endossar uma violação de independência condicional é um tanto arbitrária. Uma possibilidade é demarcar um valor (ou patamar) pré-determinado teoricamente sustentável – por exemplo, 0,20 ou 0,25 – e contrastar estatisticamente os modelos com ou sem a correlação residual livremente estimada. Outra possibilidade é seguir recomendações de autores para orientar o processo de decisão. Reeve et al.^53^sugerem a simples demarcação de ≥ 0,3 para admitir a existência de correlação residual. Há, também, demarcações baseadas em estatísticas formais. Uma é a estatística de dependência local baseada em qui-quadrado (LD χ^2^), proposta por Chen e Thissen^54,55^, que usa o corte de ≥ 10 para indicar dependências. Outra é a estatística Q3 (e variantes) sugerida por Yen^56–58^. Diversas situações levam à correlação entre resíduos (erros) de itens^59^, mas uma comum em processos de desenvolvimento (ou ATC) de instrumentos se refere à presença de redundância de conteúdo (parcial) entre itens (em geral, pares). Avaliação teórica, observando semântica e significados denotativos e conotativos dos respectivos conteúdos, deve ser realizada quando uma violação estatística é observada.
	Convergência fatorial (VFC).	Os itens, em conjunto, refletem de modo convergente o fator correspondente?	AFC. Variância média extraída.	A VFC se refere a cada fator, como o próprio nome indica. Entende-se que há VFC se a relação entre a VME dos itens – i.e., a variância que os itens têm em comum – é, ao menos, maior do que a variância conjunta dos respectivos erros – que expressam a variabilidade dos itens, que não se deve aos fatores de interesse. Assim, quantitativamente, endossa-se a VFC se a VME ≥ 0,5^17,60^. Na perspectiva interpretativa, endossando-se VFC, aceita-se que a dimensão (fator) em tela está “bem servida” pelo respectivo conjunto de itens, uma vez que estes contêm mais informatividade relativa ao fator do que mero erro (seja amostral e/ou de aferição/processo e/ou inerente aos próprios itens componentes^61^). Um indicador conexo – √ *AVE* – resume a confiabilidade do construto (dimensão). Assim, valores ≥ 0,7 também indicam convergência e, estritamente, que há consistência interna (i.e., consistência dos/entre os itens, interna ao fator a que pertencem)^60^.
	Discriminância fatorial (VFD).	A quantidade de informação captada pelo conjunto de itens em seus respectivos fatores é maior do que aquela compartilhada entre os fatores componentes (discriminante)?	AFC. Contraste da variância média extraída (pelos itens) de um determinado fator com o quadrado das correlações deste fator com os outros do sistema.	Esta propriedade somente se aplica a construtos multidimensionais. Se há VFD, espera-se que haja mais informação “fluindo” dos fatores para os itens do que entre os próprios fatores. A demarcação de violação de VFD pode seguir alguma regra genérica ( *rule of thumb* ) ou uma avaliação mais formal. Em relação à primeira, alguns autores sugerem correlações fatoriais de 0,80 a < 0,85 como suspeitos e ≥ 0,85 como indicativos de violação^17^. Uma estratégia mais rigorosa consiste em testar formalmente a significância estatística da diferença entre a VME do fator e o quadrado das correlações deste fator com os demais^60^. Um sinal positivo e estatisticamente significativo dessa diferença endossaria a VFD, ao passo que um sinal negativo estatisticamente significativo favoreceria sua rejeição, isto é, de que há uma violação. Uma diferença não significativa positiva ou negativa pode ser uma indicação a favor ou contra uma violação. Adotando-se uma posição mais conservadora, a decisão por uma violação poderia se basear somente em uma diferença estatisticamente significativa.
Escalar	Cobertura de informação do traço latente (referente a cada item e para o conjunto de itens).	O conjunto de itens cobre a maior parte do traço latente ou há regiões “desmapeadas”? Nas regiões do traço latente efetivamente mapeadas, os itens se distribuem de maneira uniforme ou há aglomerados indicando redundância de posicionamento?	TRI paramétrica. *Eyeballing* , usando o Wright Map, que consiste em combinar o mapa do construto com estimativas de posicionamento dos itens obtidas nas análises TRI e observação de gráficos.	Espera-se que os itens selecionados como manifestos do traço latente sejam capazes de adequadamente posicionar indivíduos (ou qualquer outra unidade de análise) ao longo do mapa do construto. Não menos importante, o espectro de variação previsto pelo mapa do construto também deve ser coberto de modo apropriado. Uma forma de avaliar esses dois aspectos é apreciar criticamente a disposição dos itens segundo o Wright Map proposto^13,27^. Nesse sentido, aprecia-se a correspondência do posicionamento desses itens ao longo do espectro latente – por exemplo, via parâmetros b_i_obtidos nas análises TRI – e o crescente de intensidade apresentado no mapa do construto^13^. Esse procedimento de *eyeballing* deve ser seguido de uma análise da cobertura de informação^21,62^. Gráficos específicos permitem indicar se o conjunto de itens cobre a maior parte do traço latente ou se há regiões com lacunas, “despovoadas” de itens. Esses gráficos ajudam igualmente a detectar se todas as regiões do traço latente são efetivamente abrangidas, se os itens se distribuem de maneira razoavelmente uniforme ou se há aglomerados, indicando sobreposição e, assim, redundância de posicionamento/mapeamento. Avaliações gráficas adicionais permitem, de forma complementar, apreciar o comportamento dos itens, principalmente quanto às regiões de cobertura do traço latente. Obtidos via TRI paramétrica, esses gráficos incluem as Funções de Informações dos Itens e as Curvas da Característica do Item. No caso de os itens serem policótomos, obtêm-se as Curvas da Característica de Categorias. Essas servem também para avaliar “internamente” os itens, observando-se as áreas de cobertura de cada nível e se estas estão ordenadas conforme o pressuposto teórico encerrado no mapa do construto. Exemplos destes gráficos se encontram nas referências de fundamentação indicadas ao final deste Quadro ou em buscas na internet (e.g., https://www.stata.com/manuals/irt.pdf).
	Ordenamento com escalabilidade e monotonicidade de itens.	Os itens que estão mapeando regiões do mapa do construto fazem-no na ordem de intensidade teoricamente esperada ou existem regiões do construto onde itens menos severos (mais leves/brandos) suplantam outros itens que, em princípio, deveriam capturar áreas mais intensas desse traço latente?	TRI não paramétrica e paramétrica. H de Loevinger, Critério de Mokken e avaliações gráficas.	Espera-se que os itens consigam separar bem as regiões do traço latente (conteúdo) que devem supostamente cobrir, evitando ao máximo a ocorrência de sobreposição. Duas estratégias permitem checar isso: ordenamento com escalabilidade e monotonicidade. Ordenamento de itens com escalabilidade se refere à coerência entre as frequências com que os itens são endossados e a porção do mapa do construto que deveriam teoricamente cobrir. Em um cenário ideal, espera-se que um respondente com baixa intensidade de um determinado traço latente do construto (dimensão) efetivamente endosse um item representante (mapeador) dessa região de “menor” intensidade, ao mesmo tempo que negative outro item manifestando um grau mais intenso do construto. A análise desse aspecto pode ser realizada tanto para cada item quanto para todo o conjunto do instrumento. O coeficiente de escalabilidade H de Loevinger reflete isso^63–65^. Tendo o valor 1,0 como limite superior de adequação, recomenda-se uma estimativa para o conjunto de itens de, pelo menos, 0,3^64,66^. Um H abaixo desse valor qualifica o instrumento como de má escalabilidade. Segundo Mokken^66^, valores de 0,3 a < 0,4 indicam escalabilidade fraca; de 0,4 a < 0,5, média; e ≥ 0,5, forte. Em um instrumento aceitável, espera-se igualmente que a maioria das estimativas H de cada item também siga essas referências. O pressuposto de monotonicidade é outra propriedade conexa a ser apreciada durante a avaliação do comportamento escalar dos itens e, por extensão, do conjunto formado por estes^64,65^. A monotonicidade pode ser subscrita quando a probabilidade de confirmação (positivação) de um item aumenta de maneira correspondente ao aumento de intensidade do traço latente. Visualmente, há violação de monotonicidade simples quando há declínio(s) de probabilidade de endosso à medida que cresce o escore total (latente). Adicionalmente, entende-se como violação de monotonicidade dupla se há algum cruzamento ao longo das curvas dos itens obtidas numa análise TRI. Seja simples ou dupla, considera-se que a monotonicidade está presente quando o critério sugerido por Mokken for < 40^66^, entendendo que alguns cruzamentos podem ser atribuídos à variabilidade amostral. Valores entre 40 e 80 servem de alerta, suscitando uma avaliação mais detalhada dos pesquisadores; um critério > 80 levanta dúvidas sobre a hipótese de monotonicidade de um item, bem como da escala como um todo^63,64^.

^a^Legenda: ACP – análise de componentes principais; AFC – análise fatorial confirmatória; AFE – análise fatorial exploratória; MEEE – modelos de equação estrutural exploratória; TRI – modelos de teoria de resposta ao item; VFC – validade fatorial convergente; VFD – validade fatorial discriminante; VME – variância média extraída.

^b^Referências de fundamentação: Gorsuch^67^Rummel^68^, Brown^17^, Kline^19^, Marsh et al.^48^, Embretson e Reise^62^, Bond e Fox^27^, De Boeck e Wilson^69^, Van der Linden^21^, Davidov et al.^30^Algumas destas referências são também assinaladas ocasionalmente, quando necessário, juntamente com outras específicas.

O Quadro 2 evidencia quantas propriedades necessitam ser escrutinadas antes que se possa julgar a estrutura interna como adequada e, assim, endossar este componente de validade do instrumento^15 , 16^ . É um panorama que em muito contrasta com o que a literatura habitualmente oferece, em que a validade de um instrumento tende a ser satisfeita por evidências um tanto quanto escassas e frágeis. Com efeito, não raramente decisões sobre a aceitabilidade de uma escala se escoram em algumas poucas análises fatoriais usando apenas índices de ajuste de modelo, demarcados por pontos de cortes genéricos (e.g., *Root Mean Square Error of Approximation/* RMSEA, *Comparative Fit Index/* CFI, *Tucker-Lewis Index/* TLI^17^ ) e carecendo de exames mais aprofundados sobre os itens e a(s) escala(s) como um todo. A rigor, a gama de propriedades arroladas no Quadro 2 não cabe em produtos únicos (e.g., artigos científicos), sendo necessários, para tal, estudos seriais visitando um ou mais aspectos por vez. Os meandros metodológicos relativos a cada propriedade a ser coberta certamente exigem detalhamento e maior espaço editorial.

Um ponto já abordado ilustra esse rigor fundamental: a necessidade de demarcações explícitas para se decidir se um item ou escala atende à propriedade em escrutínio. Todos os estimadores utilizados nas avaliações requerem delimitação de pontos de corte, de tal sorte que escolhas possam ser replicadas ou, se for o caso, criticadas, rejeitadas ou alteradas no decurso do desenvolvimento ou da ATC de um instrumento. O Quadro 2 procura oferecer alguns marcos indicados na literatura afim. Mais do que parâmetros de referência prescritivos, estes devem nos servir de estímulo ao exame empírico do instrumento. O ponto principal é que as muitas decisões a tomar rumo à adequabilidade psicométrica de um instrumento precisam de âncoras claras e previamente acordadas com os pares da comunidade científica. A literatura, por certo, se enriqueceria se estes pormenores se estendessem aos artigos de divulgação científica.

Uma questão a salientar é que, no contexto processual em tela, as análises multivariadas têm utilidade primordial como dispositivos diagnósticos. Sendo ferramentas de processo, devem atender às perguntas centrais postuladas *a priori* . Nesse sentido, é preciso distinguir as questões eminentemente qualitativas das quantitativas que envolvem estritamente a esfera técnico-metodológica. A terceira propriedade configural apresentada no Quadro 2 serve de exemplo. Mais do que simplesmente verificar se uma análise fatorial exploratória mostra uma carga cruzada, importa responder se efetivamente há violação de especificidade fatorial, o que seria antitético ao projetado na primeira fase, quando da elaboração do protótipo. A presença de uma carga cruzada de larga monta sugere ambiguidade no item em tela, que não seria exclusivo ao fator pressuposto e que, portanto, sua função como um “representante empírico” do mapa do construto não seria satisfeita. Aqui, a evidência quantitativa atende à qualitativa, sinalizando que há problema e necessidade de ação, seja modificando a semântica do item, seja substituindo-o por outro de melhor propriedade. Em nada diferentes, as demais propriedades demandam o mesmo olhar.

Para além das propriedades internas de itens e escalas sintetizadas no Quadro 2 , duas outras questões relacionadas merecem alusão por sua recorrência. A primeira diz respeito à presunção de invariância de medida (configural, métrica e escalar)^17^ . A suposição de que o desempenho de um instrumento não varia em domínios populacionais diferentes é quase uma regra. No mais das vezes, assume-se tacitamente que o funcionamento do instrumento é consistente entre os diversos grupos populacionais investigados (e.g., gêneros, faixas etárias, escolaridades, estratos geográficos), de modo que as diferenças encontradas entre eles são tomadas como factuais e não decorrentes de problemas de mensuração. No entanto, esta é uma posição difícil de sustentar sem maiores evidências, uma vez que o funcionamento inconsistente de um instrumento em subgrupos populacionais pode conduzir a inferências espúrias e, no limite, a decisões e ações sanitárias ineficientes ou até mesmo danosas^20^ . Há de se ter cuidado nessa direção, levando os programas de investigação sobre instrumentos de aferição um passo adiante. Não cabe apenas escrutinar suas propriedades, mas avaliá-las em diversos segmentos populacionais. Garantir invariância do instrumento em diferentes grupos populacionais é permitir comparações fidedignas.

Adjacente à invariância está a questão da equalização e ligação ( *linking* ) de instrumentos^22^ . Trata-se da busca de métricas comuns a instrumentos que supostamente captam o mesmo construto, mas que possuem itens distintos e/ou com opções de resposta variadas^25 , 26^ . Em ambas as situações há de se ter cuidado ao oferecer sínteses. Resultados de estudos podem não ser comparáveis se, mesmo focados em um mesmo construto, são realizados em domínios populacionais diferentes e com instrumentos distintos. Sem equalização, ferramentas de aferição podem carecer de sintonia métrica e escalar.

Também ligada às propriedades escalares de um instrumento está a adequação de agrupamentos quando se aplicam pontos de corte a um escore (seja bruto, formado pelo somatório dos escores dos itens componentes, seja baseado em modelos, como o são os escores fatoriais ou Rasch^27 , 28^ ). Esse ponto merece atenção, especialmente no que diz respeito às abordagens frequentemente utilizadas em epidemiologia. Não é incomum categorizar um escore em um ou poucos grupos, muitas vezes o inflexionando na média, na mediana ou em algum outro ponto “estatisticamente interessante”. Esse procedimento, no entanto, não é desprovido de riscos, uma vez que a população de estudo não necessariamente é particionada em grupos homogêneos internamente e heterogêneos entre si. O conhecimento de especialistas sobre o objeto é certamente fundamental no processo de se especificar agrupamentos adequados, mas a busca da semelhança interna de grupos com distinção comparativa pode ser mais bem servida utilizando-se adicionalmente abordagens baseadas em modelos, tais como análises de classes latentes ou modelos de mistura finita^29^ .

## AVALIAÇÃO DA CONEXÃO CONSTRUTO-TEORIA

O Quadro 3 propõe uma tipologia, na linha do que seria a validade por teste de hipótese apresentada no início da década de 2010 pela iniciativa COSMIN ( *COnsensus-based Standards for the selection of health Measurement INstruments* )^15 , 16 , 33^ . Ao contrário da concisão aparente da tipologia, esta etapa da segunda fase de avaliação de um instrumento implica, de fato, um longo processo, talvez tão longo quanto caberia estudar o próprio construto em tela, em todas as suas relações de causas e efeitos. Revisitando outros textos^7 , 11^ , valeria lembrar que determinar a validade de um instrumento corresponde, em última instância, ao estabelecimento da própria validade da teoria da qual faz parte o construto que o instrumento se propõe a medir. Um tanto circular e algo desalentador devido ao longo caminho que projeta, este raciocínio, por sua vez, nos alerta para o quão arriscado e imprudente é restringir o endosso e a aprovação de um instrumento a algumas poucas investidas de pesquisa. A solidificação e, por fim, o aval de adequabilidade de um instrumento requerem muitas testagens, seja no âmbito interno ao instrumento, seja de suas conexões externas.

**Quadro 3:** Avaliação da conexão construto-teoria.

Etapa de avaliação	Questões a serem respondidas	Técnica/método/modelo^a^	Comentários^a^
Avaliação de relações entre (sub)escalas do instrumento.	As (sub)escalas que constituem o instrumento se associam na direção e magnitude esperadas?	Testes de associação paramétricos ou não paramétricos entre as (sub)escalas que constituem o instrumento.	Esse aspecto já poderia ter sido preliminarmente contemplado na avaliação de validade discriminante envolvendo correlação fatorial, na etapa de avaliação da estrutura interna. Nesse momento de análise, porém, os testes já são baseados nos próprios escores das escalas (sejam brutos ou estimados em modelos), refinados em etapas anteriores, principalmente quanto à estrutura escalar.
Avaliação de relações entre as (sub)escalas com outros instrumentos do mesmo construto que não sejam considerados de referência	O instrumento se associa com outro que afere o mesmo construto de forma semelhante (convergente)? Com que magnitude?	Comparação de grupos extremos e testes de associação paramétricos ou não paramétricos.	Esta etapa diz respeito à validade de construto. Em conjunto, a validade de constructo, de conteúdo e de critério são conhecidas como os três Cs descritos em muitos livros-texto no âmbito da teoria clássica de medida.
Avaliação de relações entre as (sub)escalas com outro instrumento (ou procedimento) considerado de referência para o próprio construto.	O instrumento é capaz de medir o que se propõe quando há outro de referência?	Estimativa de sensibilidade, especificidade e área abaixo da curva ROC ( *Receiver Operating Characterstic* ) do instrumento, tendo como referência um critério concorrente (instrumento de referência) e/ou um desfecho futuro a ser predito.	A literatura tradicionalmente denomina esta etapa como validade de critério (um dos três Cs), subdividida em validade concorrente e validade preditiva.
Avaliação de relações entre a (sub)escala com outras que não sejam do construto em tela.	O instrumento confirma as predições e hipóteses gerais da teoria que o envolve, i.e., da sua rede nomológica? O instrumento deixa de se relacionar com outros construtos que não fazem parte da teoria geral que abrange o fenômeno de interesse?	Análises multivariadas de dados, modelos causais complexos e outras técnicas estatísticas que permitam avaliar as relações de interesse com maior rigor e precisão.	Avaliação de relações entre a (sub)escala com outras que não sejam do construto em tela.

^a^Referências de fundamentação: Streiner et al.^7^, Bastos et al.^3^, Reichenheim e Moraes^6^, Lissitz^70^, Armitage et al.^71^, Corder e Foreman^72^, Kline^19^, Little^61^, Hernán e Robins^5^, VanderWeele^35^.

Nessa direção, conforme sugere o Quadro 3 , validar externamente um instrumento vai de simples testes de associação entre as subescalas componentes até testes de intrincadas hipóteses sobre o construto e que a literatura entende como a rede nomológica das predições interligadas de uma teoria^5 , 7 , 34 , 35^ . Seja qual for o nível de complexidade da investida externa, uma pergunta que se impõe – e que frequentemente surge no âmbito das publicações científicas – é quando um estudo de validade externa de um instrumento deve ser executado, dadas as etapas a serem antes superadas para melhor conhecer seus meandros. Vale investir em projetos de pesquisa na linha do que o Quadro 3 indica sem antes ter uma mínima evidência sobre a sustentabilidade das estruturas configural, métricas e escalares do instrumento? É preciso reconhecer que correlações entre escalas (e.g., do instrumento em tela e de outras que cubram o mesmo construto) podem perfeitamente se materializar, mesmo diante de múltiplas insuficiências psicométricas de âmbito interno. O que significariam estas correlações, sabendo-se, por exemplo, que o conjunto de itens não atende satisfatoriamente aos requisitos de especificidade fatorial, validade fatorial convergente e escalabilidade? A resposta baseada na mera correlação indicaria validade externa, mas restaria perguntar “de quê?”, se a capacidade de representação do construto é falha e pouco informativa. Não há resposta clara a essas questões, mas é preciso levantá-las antes de se proceder “cegamente” a estudos de validade externa. O *timing* dessas etapas é evidentemente da alçada de cada programa de investigação, mas o ditado “quem tem pressa, come cru” serve de lembrete: pouco tempo e esforço (e recursos!) investidos em uma etapa pode ser tempo e esforço (e recursos!) dobrados em outra posterior.

## CONSIDERAÇÕES FINAIS

Com a leitura do presente artigo, deve ficar claro que o desenvolvimento de um instrumento de aferição envolve um processo extenso, compreendendo múltiplos estudos concatenados. Há de se notar que a trajetória pode ser ainda mais longa e tortuosa em se considerando os estudos de replicação ou quando certos estudos psicométricos suscitam questões que requerem respostas fundamentais que só a retomada da fase prototípica do desenvolvimento pode oferecer. Esse panorama contrasta sobremaneira com a forma como os investigadores em epidemiologia costumam abordar seus instrumentos de aferição. Como visto, ao contrário do que muitos supõem, evidências sobre a adequação de uma ferramenta de medida não se esgotam em um ou dois estudos sobre sua constituição dimensional, acompanhados, quiçá, da magnitude das cargas fatoriais encontradas. Esse alerta se estende também a acríticas análises de validade externa que, conforme mencionado na seção antecedente, requerem que a constituição interna do instrumento esteja minimamente cuidada.

E há também o desenvolvimento e o refino de versões para que as pesquisas realizadas em populações socioculturalmente distintas guardem comparabilidade e possam dialogar entre si. O processo de ATC não é menos intrincado do que o de um instrumento novo. Todas as fases e etapas se aplicam igualmente aqui. Aliás, um(a) pesquisador(a) realizando uma ATC frequentemente se depara com variadas lacunas no próprio programa de investigação original do instrumento. Por vezes, há problemas na execução dos estudos disponíveis; outras (muitas) vezes, diversas propriedades sequer foram estudadas. Nesse momento, o foco passa das equivalências ( *cf.* seção sobre Cenários de Pesquisa) para o cerne da própria estrutura do instrumento. Isso não é trivial, pois haverá sempre a ambivalência entre se tratar de um problema intrínseco da ferramenta e ser um problema no processo de ATC^6 , 11^ . Seja como for, examinar um instrumento em outro contexto sociocultural demanda ainda mais tempo e esforços. Não é para menos que muitos entendem as instâncias de ATC como mais uma etapa de validação de construto^33^ .

Uma questão que se coloca frequentemente é se todas as etapas precisam ser cumpridas para tornar o instrumento apto à utilização em pesquisa ou aplicação nos serviços de saúde. Esta é uma pergunta difícil de responder, mas alguns marcos podem nos guiar. Um já foi aventado na seção sobre as fases do processo: ter uma fase prototípica bem planejada e desenvolvida ajuda sobremaneira a obter resultados favoráveis na segunda grande fase do processo. Profundidade na primeira fase não somente contribui para se chegar a melhores propriedades psicométricas, mas também agrega eficiência, na medida em que vários problemas tendem a ser mitigados ou mesmo evitados precocemente. Cumpre lembrar que os estudos epidemiológicos na fase psicométrica, a rigor, costumam ser de grande porte e, logo, são raramente passíveis de replicações com vistas à resolução de anomalias emergentes.

Outro norte é recorrer aos fundamentos, lembrando sempre a essência de cada propriedade e o que significa sua violação. Por exemplo, sentiríamos firmeza em declarar um instrumento como válido e pronto para uso à luz de umas poucas análises fatoriais exploratórias – afirmando preliminarmente uma estrutura configural – e/ou alguns estudos correlacionando o(s) escore(s) da(s) (sub)escala(s) em teste com certas variáveis sociodemográficas – que ofereçam uma primeira evidência sobre a pertinência teórica? Dada a gama de possibilidades substantivas e processuais que visitamos, seria isto suficiente ou deveríamos adiar a utilização do instrumento e obter adicionais e diversas provas para apoiar sua validade? Reiteramos que não há resposta rápida e pronta, mas que, talvez, uma máxima possa nos ser útil à tomada de decisão: ainda que não estejamos preparados a deixar o ótimo atrapalhar o bom, ou mesmo deixar o bom atrapalhar o razoável, pode ser que valha a pena deixar o razoável atrapalhar o ruim. Embora seja uma perspectiva subjetiva – sempre a ser negociada entre pares –, se colocada em prática, possivelmente nos conduzirá a melhores instrumentos e, conforme já apontamos, a melhores resultados e comparações entre estudos ou ações de saúde.

O contínuo desenvolvimento, refinamento e adaptação de instrumentos de aferição deve ser visto como parte fundamental e integrada à pesquisa epidemiológica. A construção do conhecimento requer instrumental em patamares aceitáveis de validade e confiabilidade, à altura dos rigores comumente exigidos, por exemplo, na elaboração de desenhos de estudos e suas complexas análises. De nada adiantam meticulosidades e aprimoramentos nessas esferas se o diálogo entre as publicações e a apreciação de consistência das evidências científicas acabam falhando por conta da precariedade dos instrumentos utilizados. Sendo também produtos voltados ao uso coletivo, instrumentos de aferição demandam processos de desenvolvimento que pouco diferem dos encontrados para medicamentos ou outras tecnologias de saúde. E, como tal, merecem zelo e dedicação.
